# Persistent CD19^+^ B cell lymphopenia in critically ill COVID-19 patients 50 days after symptom onset

**DOI:** 10.3389/fcimb.2024.1488607

**Published:** 2024-11-22

**Authors:** Hui An, Ting Li, Xinyue Zhang, Hao Hu, Chen Zhang, Yongyu Wang, Shengwei Jin, Ming Li

**Affiliations:** ^1^ Department of Anesthesia and Critical Care, The Second Affiliated Hospital and Yuying Children’s Hospital of Wenzhou Medical University, Wenzhou, Zhejiang, China; ^2^ Key Laboratory of Pediatric Anesthesiology, Ministry of Education, Wenzhou Medical University, Wenzhou, Zhejiang, China; ^3^ School of Basic Medical Science, Wenzhou Medical University, Wenzhou, Zhejiang, China

**Keywords:** COVID-19, long covid, convalescence, aberrant immune-cell profiles, B cell lymphopenia, regulatory T cells

## Abstract

**Introduction:**

Long COVID (LC) poses a persistent challenge in clinical practice due to limited understanding of its etiology. LC is hypothesized to stem from aberrant immune responses in COVID-19. Vaccinations, which boost immune cells to restore function, could help ease LC symptoms.

**Methods:**

To exclude the impact of vaccination, we examined the immune cell profiles of recovering COVID-19 patients before vaccines were available. White blood cell differentials were monitored in ninety-twohealthy unvaccinated controls. Seventy-six unvaccinated COVID-19 patients were monitored upon admission and on the 50th day post-symptom onset (DPSO50). Peripheral lymphocyte subsets were analyzed using flow cytometry.

**Results:**

Mild cases showed no significant changes in lymphocyte counts or subsets from admission to DPSO50. By DPSO50, severe and critical cases showed almost complete recovery from lymphopenia, with critical cases having CD19+ B-cell counts approximately 45% lower than the mild group. Severe and critical cases exhibited reduced B-cell frequencies, with critical cases displaying around 48% higher natural killer (NK) cell counts. In mild cases, NK cell counts negatively correlated with B-cell counts (r=-0.528, p=0.02). Additionally, critical cases showed positive correlations between NK cell counts and CD4+ T-cell counts (r=0.83, p<0.01), and between NK cell counts and CD8+ T-cell counts (r=0.74, p<0.01). Severe cases demonstrated decreased counts of CD4+CD25+CD127lowFoxP3+ regulatory T-cells (Tregs), which positively correlated with B-cell counts (r=0.37, p<0.05).

**Discussion:**

Our findings indicate that aberrant immune cell profiles in COVID-19 patients change dynamically during recovery, depending on disease severity. This study suggests that convalescent patients from critical COVID-19 may experience long-lasting B-cell lymphopenia.

## Introduction

Persistent symptoms following COVID-19, regardless of the severity, are commonly referred to as Long COVID (LC). Therefore, accurately quantifying the prevalence of long COVID cases poses challenges due to the variations in methodologies and definitions, particularly in defining long COVID as the persistence of symptoms for at least 4 weeks as opposed to 12 weeks after the initial acute infection (Altmann et al., 2023). According to a meta-analysis of around 735,000 COVID-19 cases from multiple countries, 45% of individuals experienced long-lasting symptoms after 4 months, regardless of hospitalization status (O'Mahoney et al., 2023). Multiple hypotheses are being investigated for LC, some of which may be interconnected in the disease pathway. However, despite extensive COVID-19 data sets, including population-level epidemiological outcomes and lab findings, the scientific community still lacks sufficient evidence to establish definitive and mechanistic connections (Altmann et al., 2023). The persistence of the virus itself, acting as a reservoir for LC, may offer a parsimonious explanation. This proposition is reinforced by research on post-mortem tissues that identifies potential sites for persistent viral reservoirs (Stein et al., 2022). However, it should be noted that these tissues primarily come from individuals who had severe, acute, and fatal cases of COVID-19, so caution must be exercised when extrapolating to LC in general.

The varying degrees of viral infections in patients with different pre-existing conditions typically lead to diverse levels and durations of immune responses. For instance, patients with mild cases of Middle East respiratory syndrome (MERS) infection exhibited undetectable antibody responses (Choe et al., 2017). In the case of SARS-CoV-2 infection, more pronounced immune disruptions were found to be associated with severe illness (Mathew et al., 2020). During acute influenza virus infection, a prolonged immune activation in the recovery phase was linked to poorer clinical outcomes (Wong et al., 2018). The above-mentioned findings emphasize the significance of carrying out long-term and multi-time point studies to gain a comprehensive understanding of the inherent characteristics of B and T cell immune responses throughout the course of COVID-19 progression. Such studies will aid in obtaining a more precise assessment of the potential underlying mechanisms and the risk of developing LC.

During the acute phase of SARS-CoV-2 infection, a majority of COVID-19 patients experience a decrease in CD8^+^ T cell and NK cell counts, as well as lymphopenia. Meanwhile, the counts of double negative (DN, CD4^-^CD8^-^) T cells and B cells show an elevation or remain relatively unchanged (Chen et al., 2020; Guan et al., 2020; Huang et al., 2020; Jin et al., 2021; Zahran et al., 2021; An et al., 2022). As patients enter the phase of recovery or the LC period, persistent alterations in the homeostasis of T and B cells have been observed (Shuwa et al., 2021; Yang et al., 2021; Kudryavtsev et al., 2022). It is worth noting that regulatory T cells (Tregs) play a crucial role in immune tolerance, but they also contribute to the pathogenesis of various diseases, including autoimmune diseases, organ transplantation, and infections. In COVID-19 patients, the levels of Tregs tend to increase during the transition from mild to severe illness. However, as the disease progresses to a critical stage, the levels of Tregs decline (Alsalman et al., 2022). Prominently, follow-up studies on COVID-19 have demonstrated that SARS-CoV-2-specific memory B and T cell responses persist in all patients observed for 6-8 months (Sherina et al., 2021). However, it should be emphasized that individuals who experienced COVID-19 and donated plasma showed a persistent decline in the total number of B cells, which was observed at 2 months and even 8 months after being infected with the SARS-CoV-2 virus (Orologas-Stavrou et al., 2020; Kostopoulos et al., 2021).

COVID-19 vaccines have reduced infection rates, hospitalizations, and deaths. They help the immune system recognize the virus, produce antibodies or T cells, and create immune memory for future infections. In addition, vaccination lowers the risk of developing LC by about half and can reduce existing LC symptoms (Ayoubkhani et al., 2022). However, the relationship between LC and vaccination is complex. Research from the Pasteur Institute of Iran shows 26.8% of people with persistent complications experienced both LC and vaccine side effects, complicating differentiation (Sadat Larijani et al., 2024). Studying LC without the influence of vaccination would clarify its distinct impacts.

Noticeably, most follow-up studies did not differentiate between different severity levels of COVID-19, but rather analyzed them as a combined group of convalescent patients (including mild to critical cases). The discrepancy in findings may be attributed to variations in the severity of COVID-19 among the cohorts studied. Here, we investigated the dynamic changes of CD3^−^CD19^+^B cells, T cell subsets (CD3^+^CD4^+^ T cells, CD3^+^CD8^+^ T cells, DN T cells and Tregs), and CD3^−^CD16^+^CD56^+^ natural killer (NK) cells during both the acute infective and convalescent periods in patients with mild, severe, and critical COVID-19.

## Materials and methods

### Ethics statement

Ethics approval has been obtained from the Ethics Committee of Wenzhou Medical University (Ref 2020002). This study adheres to the ethical guidelines outlined in the 1975 Declaration of Helsinki, with participants providing written informed consent for sample collection.

### Study design and participants

A total of 76 unvaccinated patients in the convalescent phase (30 days after discharge) of SARS-CoV-2 infection were included in this study. These patients were among the 685 individuals diagnosed with COVID-19 and admitted to 12 hospitals in Wenzhou City, Zhejiang Province, China, between January 17, 2020, and April 24, 2020. Upon admission, COVID-19 patients were categorized into four main groups: mild cases (with mild symptoms but no radiographic features), moderate cases (fever, respiratory tract symptoms, and pneumonia visible on chest CT scan), severe cases (respiratory distress syndrome, respiratory rates ≥30/min, finger oxygen saturation measured after 5 minutes of rest ≤93%, or PaO2 [the arterial oxygen partial pressure]/FiO2 [the inspired oxygen fraction] ≤ 300 mmHg), and critically ill cases (requiring intubation due to respiratory failure, shock, other organ failures, or ICU admission). The discharged patients with COVID-19 met the following discharge criteria: being afebrile and resolution of respiratory symptoms for more than 3 days, significant improvement in lung CT images, and two consecutive negative RT-qPCR tests for viral RNA. In this longitudinal study, we included three distinct groups: a mild group (n=17), a severe group (n=53), and a critical group (n=6). The inclusion of these different groups enables us to examine the immune-cell profile of severe or critical cases, compared to a control group consisting of mild cases. This experimental design assists us in distinguishing immune-cell profiles that are specifically linked to severe or critical illness from those that might be more generalized effects of the disease. The sixth edition of the COVID-19 diagnosis and treatment plan issued by the National Health Commission was followed to identify and classify COVID-19 patients. The follow-up subjects were examined by the fourth week after discharge.

### Data collection and laboratory procedures

**Figure 1:**
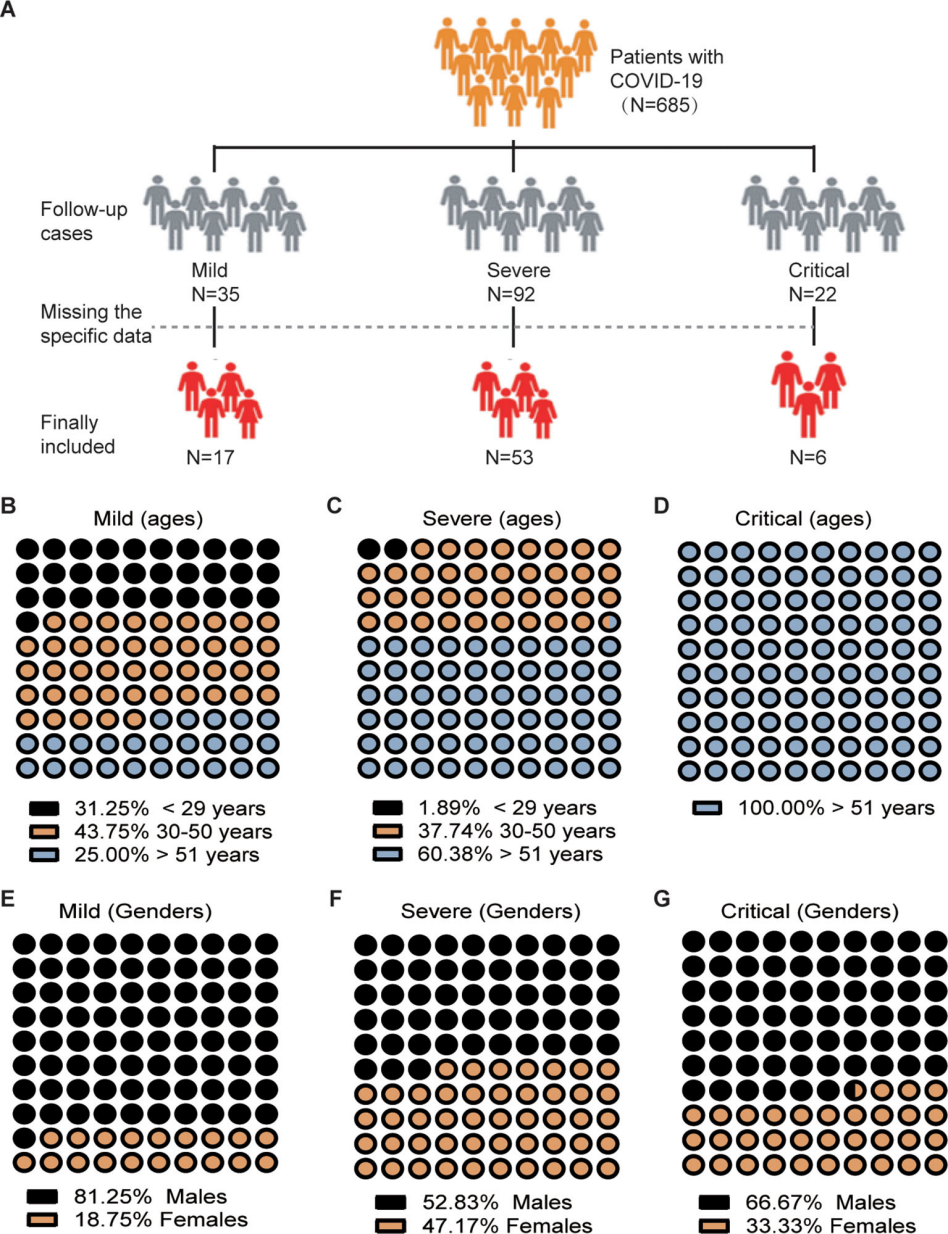
Flowchart and Demographic Characteristics of COVID-19 Patients Enrolled in the Study. **(A)** Flowchart illustrating the process of patient inclusion. **(B-G)** Distribution of patients according to age and sex.

Data on admission, including electronic medical records, epidemiological, demographic, clinical, laboratory, treatment, and outcome data, were collected using a standardized data collection form based on the WHO/International Severe Acute Respiratory and Emerging Infection Consortium case record form for severe acute respiratory infections. Ethylenediaminetetraacetic acid-anticoagulated peripheral blood samples were obtained from patients who underwent COVID-19 testing either on the day of hospitalization or on the 30th day after discharge. Routine blood examinations, such as complete blood count (including white blood cell, neutrophil, lymphocyte counts, T-cell, B cell and NK cell counts and analysis), were performed. **Figure 1A** depicts the criteria utilized for the inclusion and exclusion of the study participants. Initially, individuals diagnosed with COVID-19 were excluded from further investigation due to lack of consent from the patients. Furthermore, in some patients during the follow-up study, certain parameters such as CD8 cell or NK cell counts couldn’t be determined and were consequently absent in their records. Ultimately, a total of 76 patients who underwent follow-up investigations were included in this study, and their records were retrospectively reviewed. White blood cell differentials were monitored and data were collected from ninety-two healthy unvaccinated controls.

### Flow cytometric analysis

To analyze the phenotypic characteristics of lymphocytes (including CD4^+^ and CD8^+^ T cells, CD19^+^B cells, and NK cells), peripheral blood samples anticoagulated with ethylenediaminetetraacetic acid (EDTA) (2 mL) were collected from COVID-19 patients before initiating treatment and again after 30 days of hospital discharge. The measurement methods used were previously described (Jiang et al., 2020). In brief, staining for CD4^+^ and CD8^+^ T cells, CD3^+^CD4^-^CD8^-^ DN T cell, Tregs (CD4^+^CD25^+^CD127^low^/FOXP3^+^), CD19^+^ B cells and CD16^+^ CD56^+^ NK cells, was performed using the following antibodies: peridinin chlorophyll protein (PerCP)-conjugated anti-human CD3 monoclonal antibody (Ab), allophycocyanin (APC)-conjugated anti-human CD4 Ab, FITC-conjugated anti-CD127 Abs, PE-Cy7-conjugated anti-CD25 Ab and PE-conjugated anti-FoxP3 Ab and APC-conjugated anti-human CD19 Ab from BD Biosciences (California, USA). APC/Cy7-conjugated anti-human CD8 Ab, APC-conjugated anti-human CD16 as well as Brilliant Violet 510 (BV-510)-conjugated anti-human CD56 Ab from BioLegend (USA). The gating strategy for CD4^+^ T cells, CD8^+^ T cells, DN T cell, Tregs, CD19^+^ B cells, and NK cells was defined as CD3^+^CD4^+^, CD3^+^CD8^+^, CD3^+^CD4^-^CD8^-^, CD4^+^CD25^+^CD127^low^/FOXP3^+^, CD3^−^CD19^+^, and CD3^−^CD16^+^/CD56^+^, respectively. Flow cytometry analysis of the cells was conducted using a multicolor flow cytometry system BD FACS Canto II (BD Biosciences).

### Statistical analysis

The presented results consist of medians (IQR) or numbers (percentages), as appropriate, based on the data distribution. The comparison of distributions was conducted using the D’Agostino & Pearson omnibus normality tests. In order to address parameters that were not measured in normal controls, we utilized the normal clinical values of men and women from our hospital and compared the patient groups to these reference ranges. Consequently, the cases exceeding or falling below the normal limits for men and women were calculated and categorized as data. If the data fails to meet the assumptions of parametric statistics, the skewed quantitative data (e.g., lymphocyte counts, B cell counts, NK cell counts, and CD4^+^ and CD8^+^ T cell counts) will be subjected to analysis using a two-way non-parametric ANOVA (specifically, the Scheirer-Ray-Hare test), followed by a non-parametric Kruskal-Wallis multiple comparisons test. Categorical variables are expressed as frequency (%) and were compared using Chi-square tests or Fisher exact tests (e.g., sex and age). Spearman correlation coefficients were calculated to determine the relationships between NK cell count and other lymphocyte subsets (CD19^+^ B cell, CD4^+^, and CD8^+^ T cell counts), as well as the relationships between Tregs count and CD19^+^ B cell counts. A p-value of less than 0.05 was considered statistically significant. The data analysis was performed using SPSS software (SPSS standard, version 25.0; SPSS, Inc., Chicago, IL, USA).

## Results

### Clinical evaluation of patients

Mild COVID-19 cases had mild symptoms and no radiographic features. Comparing mild cases to healthy unvaccinated controls (NC), there were no significant differences in white blood cell, neutrophil, and lymphocyte counts between the NC and mild groups upon admission and at DPSO50 (**Table 1**). This suggests that lymphopenia does not occur in mild cases, making this group suitable as a control for analyzing severe and critical cases.

**Table 1:** White blood cell differentials in mild COVID-19 cases (Mild) on admission and DPSO50 comparable to normal controls (NC).

	NC (n=92)	Mild (n=17)		p value
Gender (male/female)	73/19	14/3		NS^*^
Age (year)	24.5 (22.0,27.0)	40.5 (26.0,49.5)		0.0003^ǂ^
BMI (Kg/m^2^)	22.30 (20.30,25.00)	22.42 (19.16,23.97)		NS^ǂ^
		Admission	DPSO50	
WBC counts (×10^9^/L)	5.77 (5.06,6.83)	5.42 (4.02,7.66)	5.52 (5.03,7.57)	
>10 (×10^9^/L)	3/92 (3.26)	0/17 (0)	1/7 (14.29)	NS^*^
Neutrophil counts (×10^9^/L)	3.26 (2.70,4.17)	3.00 (2.16,5.20)	2.90 (2.45,4.40)	
>6.3 (×10^9^/L)	4/92 (4.35)	0/17 (0)	0/7 (0)	NS^*^
Lymphocyte counts (×10^9^/L)	1.82 (1.49,2.23)	1.40 (1.08,2.15)	2.00 (1.80,2.32)	
< 1.0 (×10^9^/L)	0/92 (0)	1/17 (5.88)	0/7 (0)	NS^*^

Data presented as median (interquartile range, IQR). p values were determined using the Mann-Whitney U test (ǂ) or Fisher’s exact test (*), as applicable. NS denotes non-significant findings.

Among the 76 patients, there were 46 men and 30 women (**Figures 1E–G**), with a median age of 52 years (interquartile range [IQR] 44–62). The severity of COVID-19 is linked to age, with older individuals who have a median age greater than 51 years being more likely to develop severe or critical illness (p<0.001, p<0.01 respectively) (**Figures 1B–D**). A total of 47 (61.84%) out of the 76 patients had comorbidities, with hypertension being the most common, followed by diabetes, chronic liver disease, chronic lung disease, and chronic heart disease. Severely ill patients, upon admission, exhibited a significantly higher incidence of sputum production (p = 0.009). Three groups of COVID-19 patients, categorized as mild, severe, and critical, underwent a post-symptom onset follow-up of approximately 50 days (**Table 2**).

**Table 2:** Characteristics of enrolled patients.

	All (n=76)	Mild (n=17)	Severe (n=53)	Critical (n=6)	p value
Gender(male/female)	46/30	14/3	28/25	4/2	0.09
Age (year)	52.0(44.0,62.0)	40.5(26.0,49.5)	54.0(47.5,65.5)***	58.5(52.0,67.0)**	<0.0001
Body temperature (℃)	37.2(36.8,38.0)	36.8(36.6,37.3)	37.2(36.8,38.1)	37.8(37.3,38.2)	0.07
Systolic pressure (mmHg)	130.0(121.3,139.0)	135.0(120.5,144.5)	128.0(121.0,138.0)	137.0(125.8,157.0)	0.41
Diastolic pressure (mmHg)	81.0(74.3,89.8)	82.0(76.5,96.5)	81.0(73.5,88.0)	82.5(73.0,91.3)	0.59
Pre-existing disorders (yes/no)					
Chronic heart disease	1/75	0/17	1/52	0/6	>0.99
Diabetes	9/67	2/15	4/49	3/3**^ǂ^**	0.02
Hypertention	31/45	2/15	27/26**	2/4	0.01
Chronic liver disease	4/72	1/15	3/50	0/6	>0.99
Chronic lung disease	2/74	0/17	2/51	0/6	>0.99
Symptoms (yes/no)					
Fever	51/25	8/9	37/16	6/0	0.05
Dry cough	24/52	7/10	17/36	0/6	0.19
Fatigue	24/52	6/11	14/39	4/2	0.14
Sore throat	10/66	3/14	7/46	0/6	0.74
Runny nose	4/72	3/14	1/52	0/6	0.06
Sputum production	48/28	6/11	39/14**	3/3	0.009
Dizzy or headache	9/67	0/17	9/44	0/6	0.15
Nausea or vomiting	9/67	0/17	9/44	0/6	0.15
Myalgia	7/69	2/15	4/49	1/5	0.51
Poor apprtite	1/75	0/17	1/52	0/6	>0.99
Diarrhea	15/61	3/14	10/43	2/4	0.72
Post-symptom onset (days)	50.0(43.0,55.8)	48.0(40.5,56.5)	50.0(43.0,54.5)	57.0(48.5,58.5)	0.20

Data are median (IQR). p values were calculated by Kruskal-Wallis test, Fisher’s exact test, as appropriate. **p < 0.01; ***p < 0.001 by Fisher’s exact test or Dunn’s multiple comparisons test compared to Mild group. **^ǂ^** p < 0.05 compared to Severe group, as determined by Fisher’s exact test.

### CD19^+^ B cell count in both severe and critical cases was significantly lower

In this study, since there was no significant difference in the lymphocyte and its subclass cell (CD19^+^ B cell, CD4^+^ T cell, CD8^+^ T cell and NK cell) counts between the data obtained at admission and the data collected on the 50th day post-symptom onset (DPSO 50) in the mild group, we have designated the mild group as the control (**Table 3**). Consistent with our findings, a study conducted on a cohort of individuals recovering from mild COVID-19 demonstrated no significant differences in CD19^+^ B cell, NK cell, CD4^+^ T cell, and CD8^+^ T cell counts when compared to a control group comprising healthy individuals (Kratzer et al., 2021).

**Table 3:** Lymphocyte subsets at the first study visit and 50 days after following COVID-19 diagnosis.

Phenotype	Mild					Severe					Critical				
	n	On admission	n	DPSO 50	P- value	n	On admission	n	DPSO 50	P- value	n	On admission	n	DPSO 50	P- value
Lymphocyte count,cells/μL	17	1400(1075,2145)	13	1959(1648,2194)	0.06	51	1005(700,1295)	47	1971(1793,2181)	<0.0001	6	785(500,1278)	6	2307(1784,2572)	0.004
n(%)<1100 cells/μL^a^		4/17(23.5)		0/13(0)	0.11		30/51(58.8)		0/47(0)	<0.0001		4/6(66.7)		0/6(0)	0.06
CD19^+^B cell,cells/μL	6	249.2(114.3,341.3)	13	238.3(183.8,314.4)	>0.99	8	169.5(90.3,262.5)	48	179.8(130.9,230.4)	0.94	6	108.0(89.5,242.8)	6	130.6(94.8,188.4)	0.94
CD19^+^B cell %	6	13.5(8.4,22.0)	16	13.1(9.5,15.7)	0.82	8	22.8(13.4,39.2)	50	9.5(7.2,11.7)	0.0005	6	20.1(9.4,32.8)	6	6.8(3.8,8.1)	0.03
CD4^+^ T cell, cells/μL	8	529.5(439.3,770.8)	13	724.8(585.0,814.9)	0.13	15	433.0(193.0,505.0)	47	711.9(565.7,820.7)	<0.0001	6	227.0(135.5,345.3)	6	699.9(517.0,1221.0)	0.002
CD4^+^ T cell%	8	35.7(33.5,46.8)	15	37.0(32.7,40.6)	0.50	15	35.6(29.6,47.4)	49	34.3(30.6,41.1)	0.44	6	25.7(15.7,38.8)	6	34.4(27.7,48.0)	0.24
CD8^+^ T cell, cells/μL	8	398.0(288.5,527.0)	13	434.1(406.2,669.1)	0.19	15	179.0(149.0,471.0)	47	535.3(409.0,730.0)	<0.0001	6	94.0(46.5,138.0)	6	509.4(399.5,832.5)	0.002
CD8^+^ T cell%	8	27.1(21.5,37.4)	15	26.0(23.6,28.8)	0.80	15	26.9(15.0,34.7)	49	27.6(22.1,35.7)	0.47	6	13.0(5.6,22.9)	6	28.1(18.6,37.5)	0.13
CD4^+^/CD8^+^ ratio	8	1.5(1.1,1.6)	16	1.4(1.2,1.9)	0.83	15	1.8(1.0,2.5)	50	1.2(1.0,1.8)	0.14	6	2.7(1.1,3.7)	6	1.2(0.8,2.7)	0.18
NK cell, cells/μL	7	315.0(166.5,391.0)	16	279.3(123.5,475.4)	0.95	8	240.5(129.0,300.3)	50	371.5(207.3,574.0)	0.09	6	83.5(70.0,192.5)	6	585.3(445.5,693.2)	0.004
NK cells %	7	27.9(9.1,35.0)	14	16.5(10.2,25.9)	0.33	8	24.7(19.1,41.7)	48	20.0(11.8,29.4)	0.12	6	11.2(8.8,28.5)	6	25.5(19.2,33.1)	0.13
CD19^+^ B cell/NK cell	6	1.18(0.29,1.70)	14	0.76(0.36,1.22)	0.59	8	0.91(0.36,1.96)	48	0.39(0.23,0.82)	0.14	6	1.56(0.52,3.25)	6	0.25(0.14,0.40)	0.01
CD4^+^ T cell/NK cell	7	1.75(1.16,5.10)	13	1.77(1.57,3.27)	0.80	8	2.03(0.75,2.29)	48	1.61(1.01,2.85)	0.85	6	2.56(0.84,3.75)	6	1.63(0.79,1.95)	0.29
CD8^+^ T cell/NK cell	7	1.30(0.72,2.36)	9	1.73(0.94,2.40)	0.84	8	1.16(0.32,1.87)	48	1.43(0.78,2.72)	0.24	6	0.82(0.33,2.08)	6	1.10(0.70,1.45)	0.59
DNT cell, cells/μL	–	–	13	65.2(45.4,178.9)	–	–	–	40	62.8(33.7,85.2)	–	–	–	6	59.7(46.8,74.3)	0.52
DNT cell %	–	–	14	6.8(3.6,13.6)	–	–	–	42	4.2(3.2,6.8)	–	–	–	6	4.2(3.4,5.2)	0.30
Treg cell, cells/μL	–	–	13	84.6(70.7,125.7)	–	–	–	40	68.0(53.1,86.6)	–	–	–	6	96.5(74.1,132.1)	0.04
Treg cells%	–	–	13	7.1(5.5,8.9)	–	–	–	42	5.7(4.1,6.9)	*vs mild	–	–	6	7.1(4.9,8.8)	0.03

Data are median (IQR), n/N (%). p values were calculated by Mann-Whitney U test, Kruskal-Wallis test, χ²test, or Fisher’s exact test, as appropriate. DPSO, Days post-sympotom onset, days. DNT cell, double negative(CD3^+^CD4^-^CD8^-^) T cell. Treg cells, CD4^+^CD25^+^CD127^low/-^FoxP3^+^ regulatory T cells.

**Figure 2:**
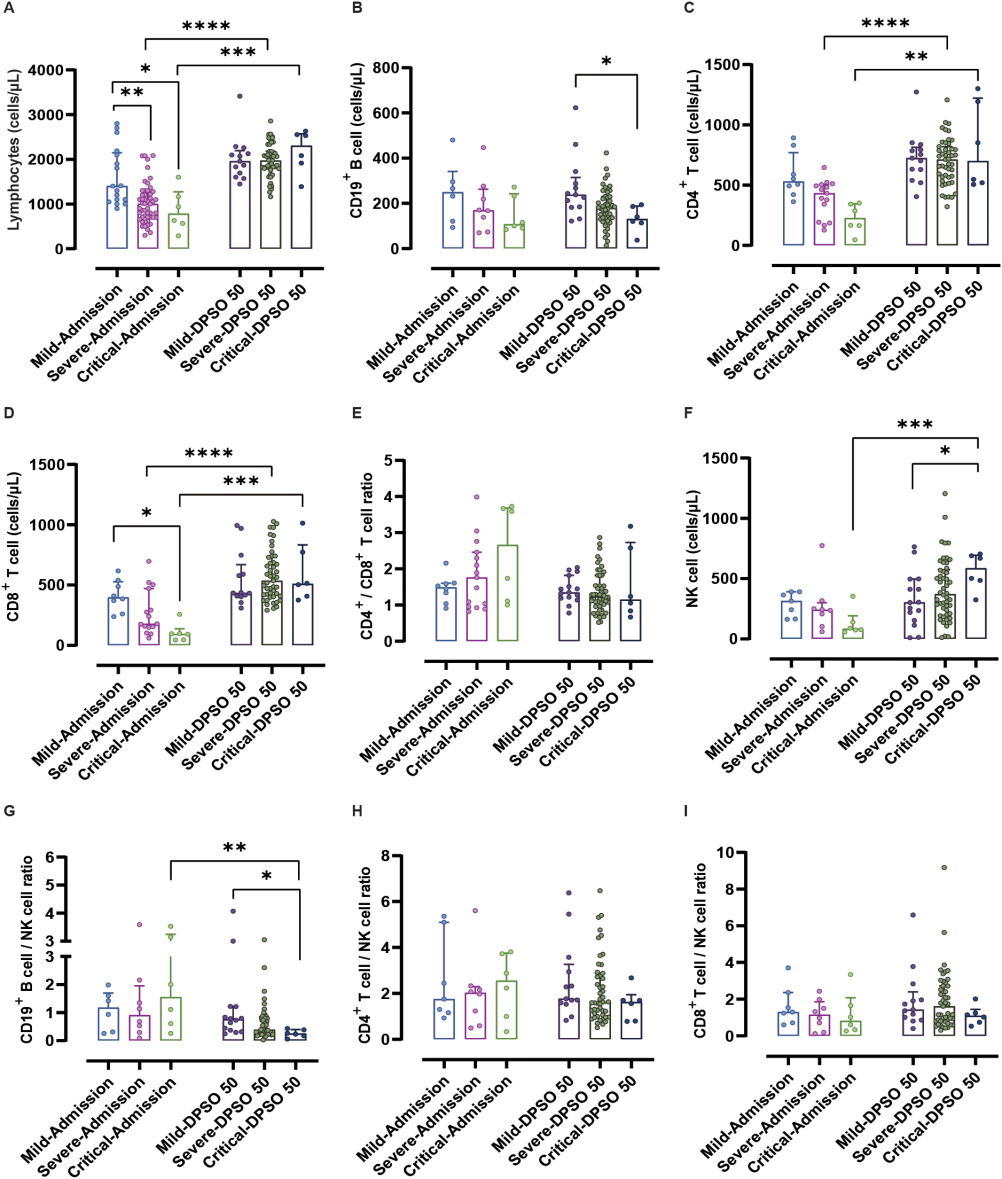
Analysis of lymphocytes and their subtypes from admission to the 50th day post-symptom onset (DPSO 50) among mild, severe, and critical groups. **(A)** Lymphocytes showed a recovery from lymphocytopenia to normal levels at DPSO 50, compared to their counts upon admission. **(B)** The count of CD19^+^ B cells in critically ill patients was significantly lower than that in mild cases at DPSO 50. **(C)** The numbers of CD4^+^ T cells and **(D)** CD8^+^ T cells in both severe and critically ill cases recovered significantly to higher normal levels at DPSO 50. **(E)** The CD4^+^ T cell/CD8^+^ T cell ratio did not significantly differ among the three groups from admission to DPSO 50. **(F)** Natural killer (NK) cell counts in critical cases were significantly higher compared to the mild cases at DPSO 50. **(G)** The CD19^+^ B cell/NK cell ratio of critical cases at DPSO 50 was significantly lower compared to the observed ratio at admission. **(H)** The CD4^+^ T cell/NK cell ratio and **(I)** CD8^+^ T cell/NK cell ratio did not significantly differ among the three groups from admission to DPSO 50. Two-way non-parametric ANOVA, followed by a non-parametric Kruskal-Wallis multiple comparisons test. *p<0.05, **p<0.01, ***p<0.001, and ****p<0.0001.

Compared to mild COVID-19 patients upon admission, severe and critical cases showed significantly decreased lymphocyte counts (p < 0.01 and 0.05, respectively). In patients with severe and critical illness, the lymphocytopenia observed at admission returned to normal levels by DPSO 50, compared to the lymphocyte count upon admission (both p < 0.001) (**Figure 2A**). No significant differences were observed in CD19^+^ B cell counts between patients with severe and critical COVID-19 upon admission, compared to mild COVID-19 patients. By DPSO 50, critically ill patients had a significantly lower CD19^+^ B cell count (p < 0.05) when compared to mild cases (**Figure 2B**).

**Figure 3:**
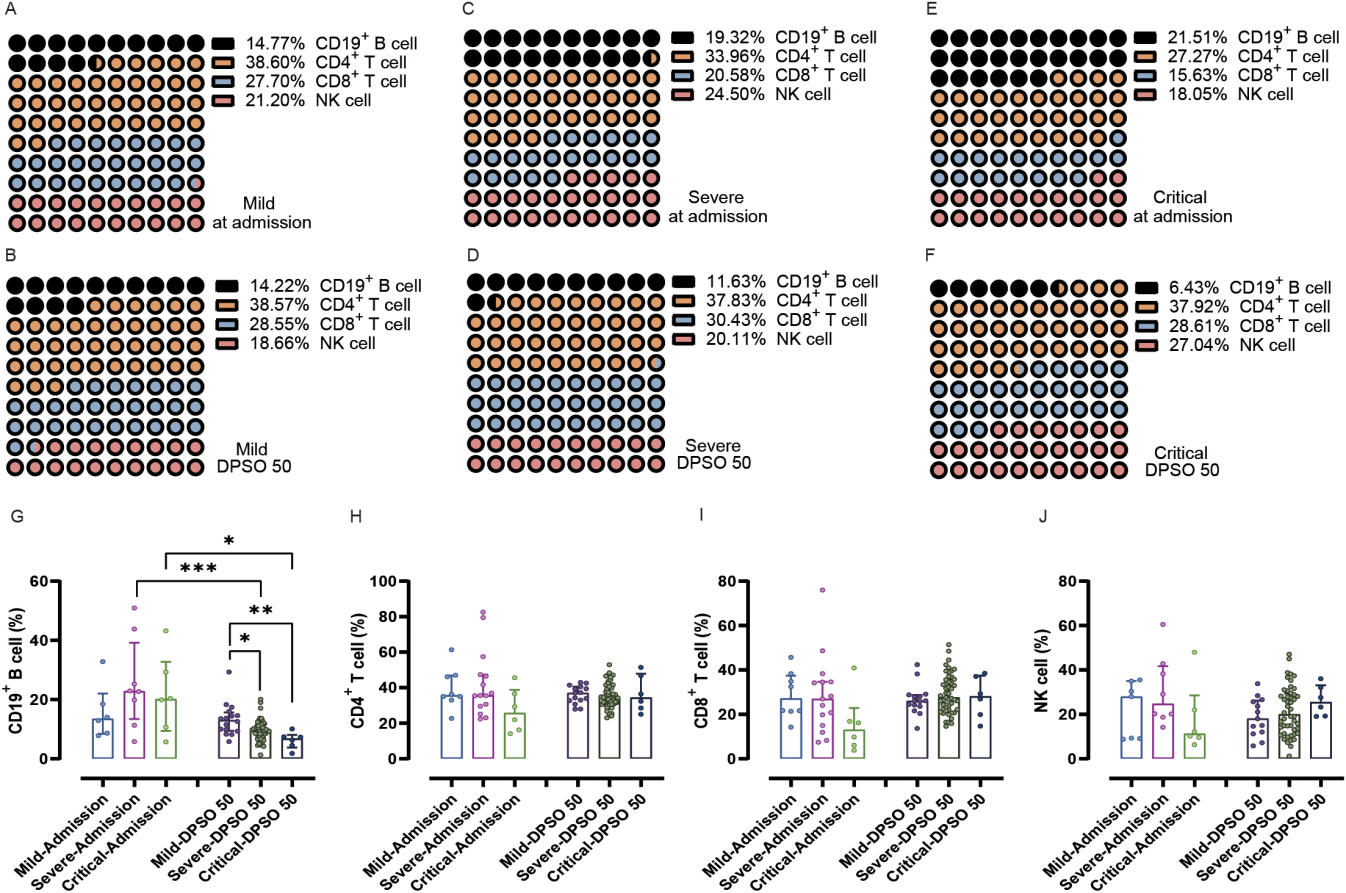
Analysis of the percentage of peripheral lymphocyte subsets from admission to the 50th day after symptom onset (DPSO 50) among mild, severe, and critical groups. **(A-F)** Dot plots (10x10) display the percentages of CD19^+^ B cells, CD4^+^ T cells, CD8^+^ T cells, and NK cells. **(G)** The percentage of CD19^+^ B cells in both severe and critical patients was significantly lower during the 50-day recovery period compared to their admission levels. **(H-J)** There were no significant differences in CD4^+^ T cells, CD8^+^ T cells, and NK cells among the three groups from admission to the DPSO 50. Two-way non-parametric ANOVA, followed by a non-parametric Kruskal-Wallis multiple comparisons test. *p<0.05, **p<0.01, and ***p<0.001.

Interestingly, the percentage of CD19^+^ B cells among total lymphocytes did not differ at admission between the mild, severe, and critical groups. However, during the 50 days of recovery, the percentage of CD19^+^ B cells in both severe and critical cases was significantly lower compared to these two groups at admission (p < 0.001 and 0.05, respectively). Furthermore, on the 50th day of recovery, there were significantly lower frequencies of CD19^+^ B cells in both severe and critical illnesses compared to the mild cases (**Figures 3A–G**).

### There are significant fluctuations in the counts and frequencies of CD4^+^ T cells, CD8^+^ T cells, DN T cells, and Tregs

There were no noteworthy differences in CD4^+^ T cell counts between mild and severe cases upon admission. However, a significant recovery to higher normal levels was observed on DPSO 50 for both severe and critical cases (p < 0.001 and 0.01, respectively; see **Figure 2C**). This indicates that viral infection indeed leads to a decrease in CD4^+^ T cell count upon admission.

Upon admission, critical cases show a significant decrease in CD8^+^ T cell count compared to mild cases (p < 0.05). There are no notable differences in CD8^+^ T cell counts between mild and severe cases upon admission. However, on DPSO 50, severe and critical cases demonstrate a significant recovery, reaching higher normal levels (p < 0.0001 and 0.001, respectively; **Figure 2D**).

**Figure 4:**
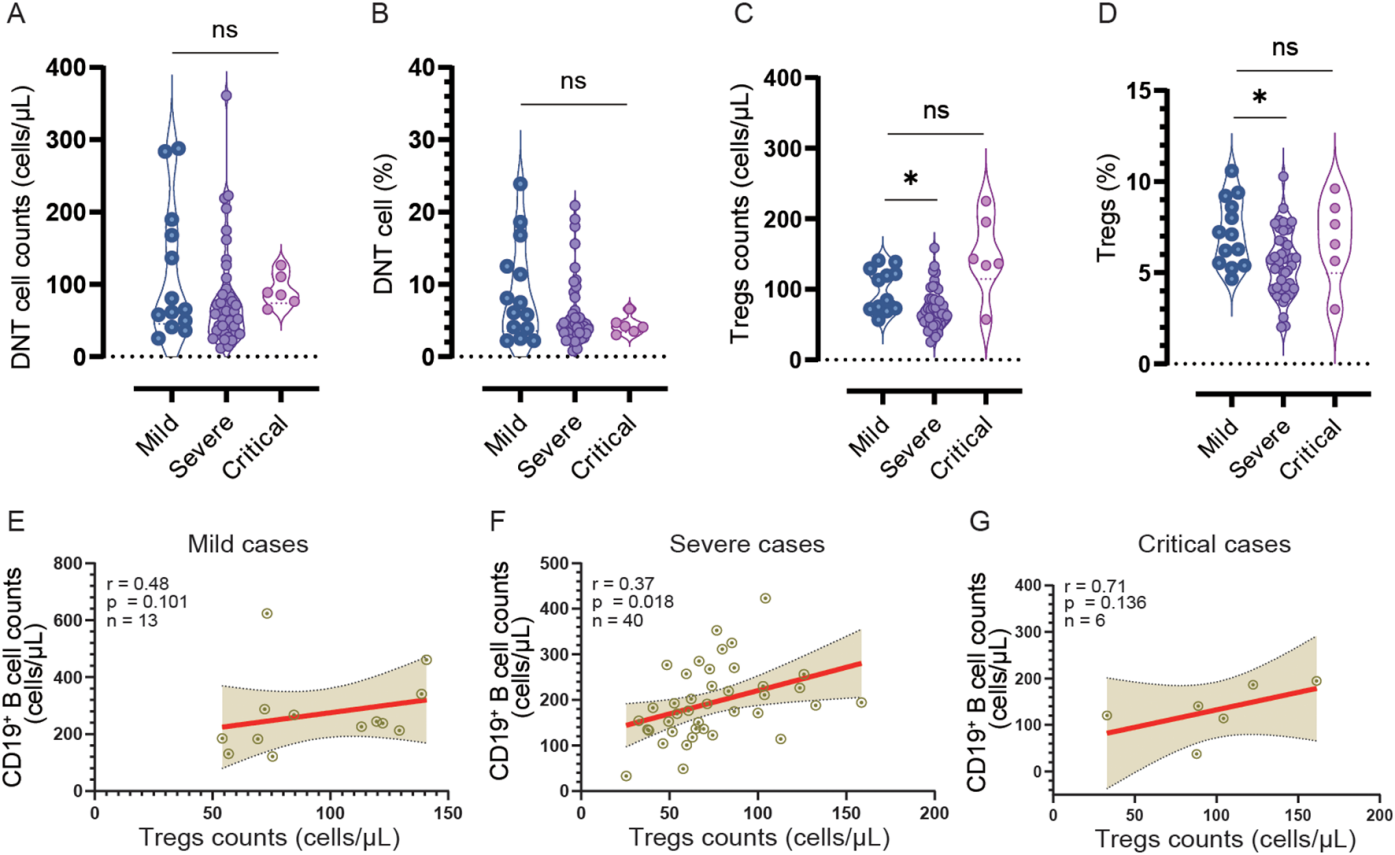
The counts and frequencies of double negative T cells (DNT) and regulatory cells (Tregs), as well as the correlation between Treg and CD19^+^ B cell counts, were analyzed in the mild, severe, and critical groups at DPSO 50. **(A, B)** There were no significant differences observed in both the counts and percentages of DNT cells among the three groups. **(C, D)** In the severe group, both the count and percentage of Tregs were significantly lower compared to the mild group. The statistical analysis was conducted using a non-parametric Kruskal-Wallis test, followed by Dunn’s multiple comparisons test. The significance level was set at p<0.05. **(E-G)** A correlation analysis was performed to investigate the relationship between CD19^+^ B cell counts and Treg cell counts separately in the mild, severe, and critical cases. Spearman correlation analysis was utilized for this analysis *p<0.05; ns, no significant.

There were no significant differences observed in the CD4^+^ T cell/CD8^+^ T cell ratio, CD4^+^ T cell percentage, and CD8^+^ T cell percentage at admission and DPSO 50 (**Figures 2E**, **3–F, H, I**). No differences in DN T cell counts and proportions were observed between mild, severe, and critical groups at DPSO 50 (**Figures 4A, B**). Compared to mild cases, a significant decrease in Tregs counts and frequencies was observed in severe cases, but the same difference was not observed in critical cases (**Figures 4C, D**).

### Notable fluctuations in NK cell counts and the proportions of CD19^+^ B cells, CD4^+^ T cells, and CD8^+^ T cells

Upon admission, there were no statistical differences in NK cell counts among mild, severe, and critical cases. However, at the DPSO 50 time point, the counts of NK cells in critical cases significantly increased compared to both the critical group at admission and the mild group at DPSO 50 (p < 0.001 and <0.05, respectively; **Figure 2F**). Similarly, as depicted in **Figure 3J**, there were no significant differences in the proportions of NK cells among lymphocytes for mild, severe, and critical cases at both the admission and DPSO 50 time points.

To assess the impact of NK cells on eliminating virus-infected immune cells, we examined the proportions of CD19^+^ B cells, CD4^+^ T cells, and CD8^+^ T cells in relation to NK cells. Among patients in critical condition, only the CD19^+^ B cell/NK cell ratio exhibited a significant decrease at the DPSO 50 compared to the ratios observed upon admission (p < 0.01). Besides, upon DPSO 50, critical cases show a significant decrease in CD19^+^ B cell/NK cell ratio compared to mild cases (p < 0.05; **Figure 2G**). However, there were no statistically significant differences between admission and the DPSO 50 in the CD4^+^ T cell/NK cell and CD8^+^ T cell/NK cell ratios for cases classified under mild, severe, and critical severity (**Figures 2H, I**).

### Correlation analysis of NK cell and Tregs counts with CD19^+^ B cell, as well as NK cell count with CD4^+^ and CD8^+^ T cell counts

**Figure 5:**
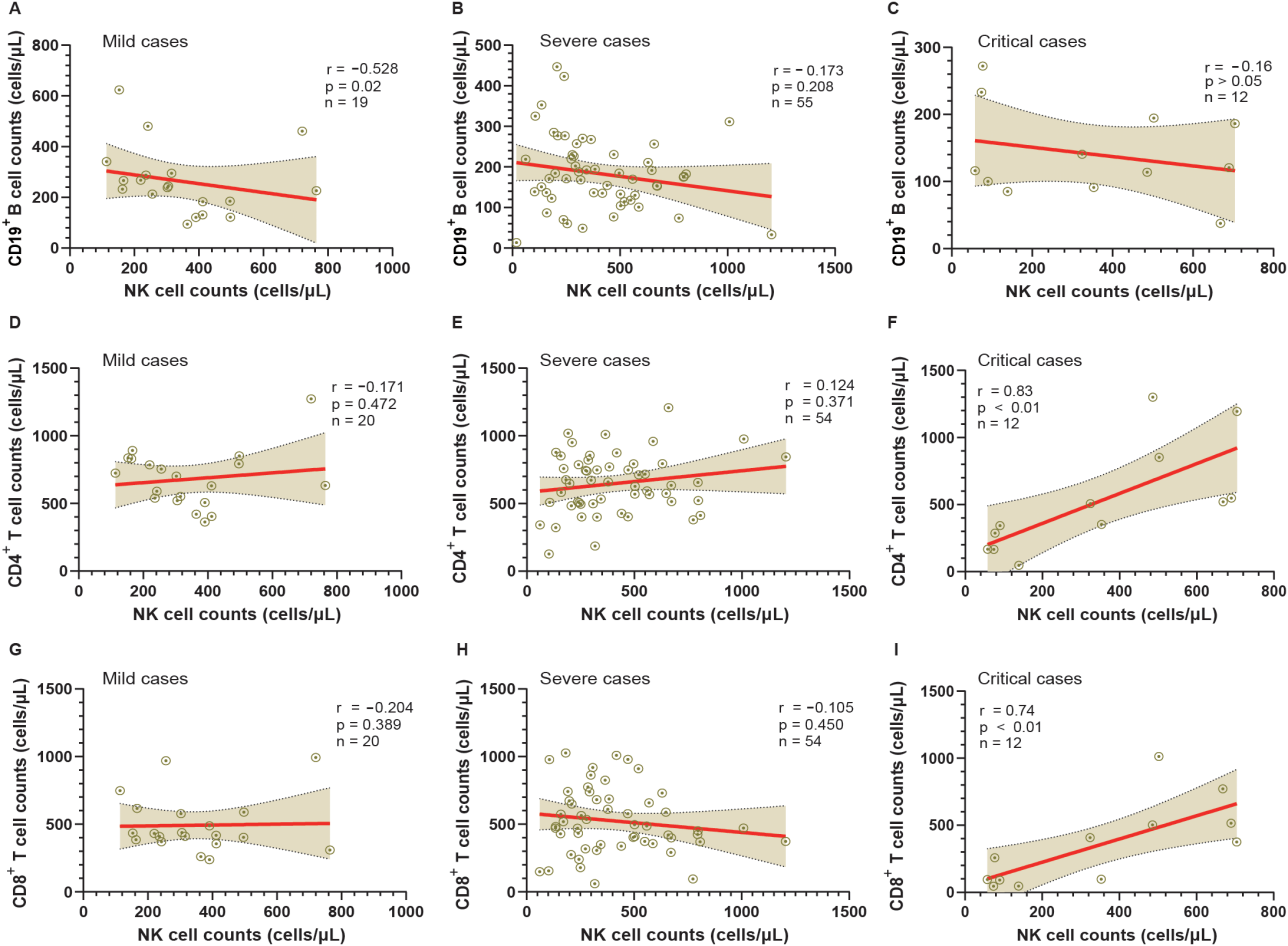
Analysis of the relationship between NK cells and other lymphocyte subtypes in mild, severe, and critical groups from admission to the 50th day post-symptom onset (DPSO 50). **(A-C)** Correlation analysis between CD19^+^ B cell counts and NK cell counts in mild, severe, and critical cases, respectively. **(D-F)** Correlation analysis between CD4^+^ T cell counts and NK cell counts in mild, severe, and critical cases, respectively. **(G-I)** Correlation analysis between CD8^+^ T cell counts and NK cell counts in mild, severe, and critical cases, respectively. Correlation was assessed using Spearman’s rank correlation analysis.

To investigate the relationship between NK cells and other lymphocyte subtypes, we conducted a comprehensive analysis examining their correlations with CD19^+^ B cells, CD4^+^ T cells, and CD8^+^ T cells (**Figures 5A–I**). Our findings reveal that in cases of mild severity, there is a significant negative correlation between the counts of NK cells and CD19^+^ B cells (r=-0.528, p<0.05). However, although negative correlations are also observed in severe and critical cases, they do not reach statistical significance (r=-0.173 and r=-0.16, both > 0.05).

In severe cases, there is a notable positive correlation between the counts of Tregs and CD19^+^ B cells (r=0.37, n=40, p<0.05). Nevertheless, in mild and critical cases, despite positive correlations being present, they fail to reach statistical significance (r=0.48, n=13 and r=0.71, n=6 respectively, both p > 0.05) due to the smaller sample sizes (**Figures 4E–G**).

Conversely, a positive correlation between the counts of NK cells and CD4^+^ T cells is solely observed in critical cases (r=0.83, p<0.01). However, no significant correlations are found between the counts of NK cells and CD4^+^ T cells in mild cases (r=-0.171, p > 0.05) and severe cases (r=0.124, p > 0.05). Moreover, regarding the correlation between the counts of NK cells and CD8^+^ T cells, no significant correlations are observed in mild cases (r=-0.204, p > 0.05) and severe cases (r=-0.105, p > 0.05). Nonetheless, a strong correlation is detected between the counts of NK cells and CD8^+^ T cells in critical cases (r=0.74, p<0.01).

## Discussion

Longitudinal studies investigating the natural course of COVID-19 in unvaccinated individuals with mild, severe and critical illnesses provide valuable insights into the dynamics and longevity of immune responses. The present study highlights several key aspects of CD19^+^ B cell, CD4^+^ and CD8^+^ T cell, DN T cells, Tregs and NK cell responses to SARS-CoV-2 infection during the acute phase and convalescent period. We observed a substantial decline in CD19^+^ B-cell count on the DPSO 50 in COVID-19 cases with critical illness, enduring for around 50 days regardless of the recovery of clinical symptoms and disease progression. However, the lymphocyte count rebounds and normalizes during this timeframe in these critical cases. This aligns with previous research indicating that although lymphopenia was completely reversed two months after infection, the proportion of total B cells did not return to normal levels even after eight months of SARS-CoV-2 infection in 27 plasma donors (Kostopoulos et al., 2021). Interestingly, a study conducted an analysis on two mixed groups of rehabilitation patients (mild+moderate group and severe+critical group), in which approximately 30% of the participants reported long-term symptoms resulting from COVID-19. The findings of the study revealed a significant increase in the percentage of CD19^+^ B cells at 12 and 16 weeks after infection, as compared to the healthy control group (Ryan et al., 2022). The disparities identified in the aforementioned studies may be partially ascribed to variances in the composition of patient groups or variations in the durations of recovery.

Our investigation revealed a significant inverse relationship between the counts of NK cells and CD19^+^B cells upon admission in mild patients affected by SARS-CoV-2 infection. Conversely, such correlation was not observed in critically ill individuals at admission, suggesting that these patients exhibited an impaired capacity to eliminate virus infected B cells due to concurrent NK cell reduction. The aforementioned findings indicate that CD19^+^ B cells have the potential to be selectively suppressed within the subset of lymphocytes in hosts infected with SARS-CoV-2. This phenomenon could potentially contribute to their ability to elude elimination of virus by the host’s adaptive immune system.

It was observed that the counts of Tregs showed a significant decrease only in severe cases, rather than in critical cases, when compared to the mild groups on DPSO 50. However, both severe and critical patients showed a decrease in B cell frequencies. Moreover, there was a notable positive correlation between the count of Tregs and the count of B cells in severe cases, while this correlation coefficient was larger in critical cases, although it did not reach statistical significance due to the limited sample size in this group (r=0.71, p=0.136, n=6). These findings suggest a potential association between the decrease in Treg counts in severe cases on DPSO 50 and changes in B cell frequencies. It is important to acknowledge that these findings are based on a limited sample size, but they suggest the possibility of an interplay between Tregs and B cells in the development of severe cases. To validate and understand the underlying mechanisms involved, further investigation with larger sample sizes is required.

According to previous studies, it has been reported that Tregs are upregulated during the acute phase of COVID-19, with an increase in severity of the disease. However, in critical cases, there is a subsequent downregulation of Tregs (Wang et al., 2020). Therefore, the regulation of Tregs in severe patients exhibits a dynamic response, characterized by an initial upregulation during the acute phase followed by a subsequent downregulation during the recovery phase. Based on the majority of studies, it is evident that Treg cells in COVID-19 patients are actively functioning, suggesting a negative feedback mechanism to protect self-tissues from immune cell damage (Wang et al., 2021). Since Tregs play a crucial role in maintaining immune homeostasis, it is commonly observed that alterations in the distribution and behavior of both Tregs and B cells are closely associated with the progression of autoimmune disorders (Azizi et al., 2018), it is plausible to consider that individuals experiencing Long COVID, which is characterized by a significant decrease in circulating B cells, may harbor serum containing anti-lymphocytic antibodies induced by the SARS-CoV-2 virus. These antibodies are believed to exhibit notable specificity towards B lymphocytes and potentially contribute to the observed B-cell deficiency in this patient population. Therefore, it is advisable to initiate comprehensive investigations aiming to elucidate the presence and functional implications of these antibodies in Long COVID patients with B-cell depletion. Such investigations could provide valuable insights into the pathogenesis of Long COVID and pave the way for potential therapeutic strategies.

A potential alternative hypothesis regarding the possible reservoirs of the SARS-CoV-2 virus within the bodies of patients with critical COVID-19 is its potential presence in the tissue of the bone marrow, specifically in the sinus of the marrow. This presence has the potential to cause harmful effects on the cells responsible for B cell proliferation and development. This conjecture is supported by several lines of evidence. Hematopoietic stem cells (HSCs), which are resident cells in the bone marrow, express a relatively high level of angiotensin-converting enzyme 2 (ACE2) receptors (Ropa et al., 2021), to which the Spike (S) protein of SARS-CoV-2 binds, facilitating viral entry. Studies have reported significant alterations in hematopoietic stem and progenitor cells caused by COVID-19. Severe COVID-19 patients exhibit a notably lower percentage of HSCs compared to non-severe cases (Hussein et al., 2023), indicating a higher virus infection of these cells in the severe patients. Furthermore, hemato-oncology patients have shown prolonged persistence of SARS-CoV-2 viral RNA (Hus et al., 2021), which can lead to chronic infection or immune exhaustion, along with associated consequences. Certainly, it is imperative to conduct a rigorous research to investigate the presence of the virus in the bone marrow of individuals suffering from Long COVID-19.

A significant increase in the number of NK cells among COVID-19 patients with critical illness during their recovery phase was noted. We observed a significant increase in the number of NK cells among COVID-19 patients with critical illness during their recovery phase. This finding suggests a potential association between the development of Long COVID-19 and elevated NK cell levels in these individuals. A recent study conducted by Galán et al. reported higher levels of NK cells in the blood of individuals with Long-COVID syndrome compared to those who had recovered. The authors of the study proposed that Long-COVID syndrome could be attributed to a persistent memory cytotoxic immune response triggered by SARS-CoV-2 or the presence of hidden viral components in specific anatomical locations (Galan et al., 2022).

Additionally, it was observed that the counts of CD4^+^ T cells and CD8^+^ T cells have returned to normal levels in severe and critical cases of COVID-19 during the rehabilitation period. This finding is consistent with previous research studies (Ryan et al., 2022), suggesting a positive progress in the immune system. Interestingly, we also noticed strong positive correlations between NK cell counts and both CD4^+^ T cell and CD8^+^ T cell counts in critical cases of COVID-19. This observation indicates a potential synergistic interaction between NK cells, CD4^+^ T cells, and CD8^+^ T cells in critically ill individuals with COVID-19. These findings suggest the presence of a unique immune cell microenvironment within these patients, highlighting the complex dynamics of the immune response during severe COVID-19 illness. However, there is an alternative perspective to understand the strong correlations between NK cells, CD4^+^ T cells, and CD8^+^ T cells. It has been commonly observed in literature that NK cells and T cells have suppressive effects on each other (Gasteiger and Rudensky, 2014). For instance, activated NK cells can eliminate both activated CD4 subsets and CD8^+^ T cells *in vitro* and *in vivo* (Daniels et al., 2020). Therefore, it is possible that during the recovery period of individuals with critical illness from COVID-19, the increase in the cell numbers of NK cells, CD4^+^ T cells, and CD8^+^ T cells could be facilitated by a common lymphoid progenitor in the bone marrow, which gives rise to precursor cells of T cells, NK cells, and innate lymphoid cells (Gasteiger and Rudensky, 2014). This suggests a potential mechanism for the simultaneous recovery of NK cells, CD4^+^ T cells, and CD8^+^ T cells in critically ill individuals, indicating a coordinated immune response.

One notable limitation of our study was the relatively small sample size and short follow-up period, especially when considering patients with critical illness associated with Long COVID-19. In order to more comprehensively evaluate the differences attributable to disease severity, treatment, and other confounding factors, larger-scale studies with longer-term follow-up will be essential. Furthermore, such studies will be crucial for validating the observed changes in immune cell profiles. Although our study provides a detailed and multifaceted understanding of lymphocyte subtype cell profiles in post-COVID-19 individuals, it is important to acknowledge certain significant limitations. Further analysis methodologies focusing on immune cell subtypes will offer a higher level of resolution in assessing the immune dysregulation experienced by convalescents and the specific cell signatures associated with long COVID.

## Conclusion

In summary, this research has discovered enduring alterations to the peripheral immune system in individuals who have recovered from SARS-CoV-2 after experiencing critical illness. These changes can be observed for at least 50 days after infection and are specifically associated with significant modifications in B cells, NK cells, CD4^+^ T cells, CD8^+^ T cells and Tregs. The interactions between these immune cell populations may play a role in the development of long COVID-19. The implications of these enduring immune system alterations are significant. Firstly, the presence of B cell lymphopenia symptoms during the convalescent period may potentially serve as a diagnostic indicator for Long COVID. Secondly, the abnormal profiles of immune cells not only affect the response of individuals recovering from severe SARS-CoV-2 illness to subsequent infections during this period, but also have the potential to exacerbate pre-existing chronic conditions as a result of prolonged immune activation. Thirdly, it is essential to recognize the significance of these findings in order to understand the long-term impact of COVID-19 on the immune system and to facilitate effective management and treatment strategies for individuals in recovery.

## Data Availability

The datasets generated during and/or analyzed during the current study are available from the corresponding author on reasonable request.
